# The ferroxidase LPR5 functions in the maintenance of phosphate homeostasis and is required for normal growth and development of rice

**DOI:** 10.1093/jxb/eraa211

**Published:** 2020-07-03

**Authors:** Hao Ai, Yue Cao, Ajay Jain, Xiaowen Wang, Zhi Hu, Gengmao Zhao, Siwen Hu, Xing Shen, Yan Yan, Xiuli Liu, Yafei Sun, Xiaoxia Lan, Guohua Xu, Shubin Sun

**Affiliations:** 1 State Key Laboratory of Crop Genetics and Germplasm Enhancement, Key Laboratory of Plant Nutrition and Fertilization in Low-Middle Reaches of the Yangtze River, Ministry of Agriculture, Nanjing Agricultural University, China; 2 School of Environmental Science and Engineering, Guangdong Provincial Key Lab for Environmental Pollution Control and Remediation Technology,Sun Yat-sen University, Guangzhou, China; 3 Amity Institute of Biotechnology, Amity University Rajasthan, Jaipur, India; 4 Landscape Architecture Department, College of Horticulture, Nanjing Agricultural University, China; 5 Institute for Plant Genomics and Biotechnology, Texas A&M University, College Station, TX, USA; 6 Institute of Eco-Environment and Plant Protection, Shanghai Academy of Agricultural Sciences, Shanghai, China; 7 Bielefeld University, Germany

**Keywords:** Agronomic traits, ferroxidase activity, growth and development, *Oryza sativa*, *LPR5*, phosphate homeostasis, rice

## Abstract

Members of the *Low Phosphate Root* (*LPR*) family have been identified in rice (*Oryza sativa*) and expression analyses have been conducted. Here, we investigated the functions of one of the five members in rice, *LPR5*. qRT-PCR and promoter–*GUS* reporter analyses indicated that under Pi-sufficient conditions *OsLPR5* was highly expressed in the roots, and specific expression occurred in the leaf collars and nodes, and its expression was increased under Pi-deficient conditions. *In vitro* analysis of the purified OsLPR5 protein showed that it exhibited ferroxidase activity. Overexpression of *OsLPR5* triggered higher ferroxidase activity, and elevated concentrations of Fe(III) in the xylem sap and of total Fe in the roots and shoots. Transient expression of OsLPR5 in *Nicotiana benthamiana* provided evidence of its subcellular localization to the cell wall and endoplasmic reticulum. Knockout mutation in *OsLPR5* by means of CRISPR-Cas9 resulted in adverse effects on Pi translocation, on the relative expression of *Cis-NAT*_*OsPHO1;2*_, and on several morphological traits, including root development and yield potential. Our results indicate that ferroxidase-dependent *OsLPR5* has both a broad-spectrum influence on growth and development in rice as well as affecting a subset of physiological and molecular traits that govern Pi homeostasis.

## Teaser

LPR5 functions as a ferroxidase and potentially affects *OsPHO1;2*-mediated acquisition and/or mobilization of phosphate in rice, thereby influencing growth and development.

## Introduction

Rice (*Oryza sativa*) is a staple and main source of dietary energy supply for ~3.5 billion people in the world, and most (~90%) is consumed in Asia alone (www.irri.org/rice-today). Increasing the productivity of rice is therefore critical for global food security. Rice ecosystems, particularly acid upland soils, often experience severe deficiency of inorganic phosphate (Pi) due to high soil P-fixation (Gamuyao *et al.*, 2012). Pi, one of the major essential macronutrients, is a key structural component and is required for several metabolic pathways (López-Arredondo *et al.*, 2014). Low Pi availability results in adverse effects on growth and development, and hence the yield potential, of agronomically important crop species (Veneklass *et al*., 2012). Pi deficiency triggers an array of morpho-physiological and adaptive molecular responses in rice (Torabi *et al.*, 2009; Oono *et al.*, 2013; Secco *et al.*, 2013; Wu *et al.*, 2013; Mehra *et al.*, 2015; Negi *et al.*, 2016; Xu *et al.*, 2016).

The root system plays a critical role in the uptake of nutrients and exhibits extensive developmental plasticity in response to different nutrient deficiencies (Gruber *et al.*, 2013; Kellermeier *et al.*, 2014). In Arabidopsis, the cells of the primary root tip have an inherent ability to sense local Pi availability independent of the whole-plant Pi status (Linkohr *et al.*, 2002), and inhibition of primary root growth is a hallmark of the Pi-deficiency response (Williamson *et al.*, 2001; López-Bucio *et al.*, 2002). Seedlings of transgenic Arabidopsis expressing the cell-cycle marker *CycB1;1::uidA* exhibit a progressive loss of its expression in the primary root tip when deprived of Pi, which triggers a shift from an indeterminate to an irreversible determinate developmental program (Sánchez-Calderón *et al.*, 2005). *Low Phosphate Root1* (*LPR1*; At1g23010), which is negatively regulated by *Phosphate Deficiency Response 2* (*PDR2*), encodes multi-copper oxidase and is associated with the inhibition of primary root growth that is mediated by Pi deficiency (Reymond *et al.*, 2006; Svistoonoff *et al.*, 2007; Ticconi *et al.*, 2009). It has been shown that root meristem-specific *LPR1* in Arabidopsis encodes a cell wall-targeted ferroxidase, which is crucial for local Pi sensing (Müller *et al.*, 2015). This study also demonstrated cell-specific expression of *pLPR1::GUS*, which was correlated with the patterns of iron (Fe) and callose deposition in the root tips of Pi-deprived wild-type seedlings. During Pi deficiency, the CLE14 peptide is expressed in the proximal meristem and is recognized by the receptors CLV2 and PEPR2, which triggers terminal differentiation of the surrounding root apical meristem, a phenomenon known as root meristem exhaustion or determined growth (Gutiérrez-Alanís *et al.*, 2017). However, a recent study has shown that it is actually the blue-light effect in the Petri dish that triggers the Fe-dependent Pi deficiency-mediated inhibition of primary root growth observed in Arabidopsis (Zheng *et al.*, 2019).

Unlike Arabidopsis, the growth of roots in rice is either stimulated (Dai *et al*., 2012, 2016) or remains unaffected during Pi deficiency (Hu *et al.*, 2011; Wang *et al.*, 2014). Although several genes associated with the embryonic and post-embryonic development of rice roots have been identified (Mai *et al.*, 2014; Wu and Cheng, 2014), the role of homologs of *LPR1* remains elusive. In a previous study, we identified five homologs of *LPR1* in rice (*OsLPR1–5*), of which *OsLPR5* displayed strong tissue-specific induction during Pi deficiency (Cao *et al.*, 2016). However, it is not known whether *OsLPR5* confers ferroxidase activity and if it affects the responses of different root traits in seedlings during growth under different Pi conditions, and/or other morpho-physiological and molecular traits that govern the maintenance of Pi homeostasis.

Here, we show that Pi deficiency-induced *OsLPR5* is a ferroxidase. Transient expression analysis indicated the subcellular localization of OsLPR5 to be the endoplasmic reticulum (ER) and the cell wall. In addition, overexpression and CRISPR-Cas9-mediated mutation of *OsLPR5* provided empirical evidence for its broad-spectrum role in the developmental responses and maintenance of Pi homeostasis.

## Materials and methods

### Plant materials and growth conditions

Rice seeds (*Oryza sativa* L. ssp. *japonica* cv. Nipponbare) were surface-sterilized for 30 min with diluted NaClO (1:3, v/v), followed by thorough rinsing for 30 min with deionized water. Seeds were germinated in theb dark at 25 °C for 3 d. Hydroponic experiments were carried out in a growth room with a 14/10-h light/dark photoperiod at 30/22 °C at 200 μmol m^–2^ s^–1^, and the relative humidity was maintained at ~70%. The containers used for the hydroponic experiments were all painted black to exclude light from the roots. Seedlings at 10 d old were transferred to a nutrient medium consisting of 1.25 mM NH_4_NO_3_, 1 mM CaCl_2_, 1 mM MgSO_4_, 0.5 mM Na_2_SiO_3_, 0.35 mM K_2_SO_4_, 0.2 mM KH_2_PO_4_, 20 μM Fe-EDTA, 20 μM H_3_BO_3_, 9 μM MnCl_2_, 0.77 μM ZnSO_4_, 0.39 μM Na_2_MoO_4_, and 0.32 μM CuSO_4_. Hereafter, this medium is referred to as +P (i.e. Pi-sufficient). For the Pi-deficient medium (–P), KH_2_PO_4_ was removed and replaced with 0.2 mM KCl. For pot experiments, the soil was obtained from an experimental farm at Nanjing Agricultural University. Each pot was filled with 15 kg of air-dried soil and supplemented with 40 mg of Pi fertilizer kg^–1^ soil. The plants were cultivated in a greenhouse at ambient temperatures and under natural daylight, as described previously (Jia *et al.*, 2011).

### Construction of promoter–GUS fusion and overexpression lines

A fragment (2289 bp) upstream of the coding sequence of *OsLPR5* was amplified from the genomic DNA of the wild-type (Nipponbare) using *OsLPR5*-specific primers. The *Bam*HI and *Kpn*I restriction sites were incorporated into these primers to facilitate directional cloning in the expression vector 1300GN. For overexpression, the coding sequence (1914 bp) of *OsLPR5* was amplified from cDNA isolated from the wild-type using *OsLPR5*-specific primers. The PCR product was cloned downstream of the ubiquitin promoter of the pTCK303 vector, digested with *Bam*HI and *Spe*I, and transformed into *Agrobacterium tumefaciens* strain EHA105 through electroporation. Both the constructs were transformed into the wild-type as described by Upadhyaya *et al.* (2000). The three independent overexpression lines generated are hereafter referred to as *Ox3*, *Ox6*, and *Ox7.* The primers used for promoter–GUS fusion and overexpression are listed in Supplementary Table S1 at *JXB* online.

### GUS histochemical analysis

Samples of panicles, nodes, and culms were collected from 17-week-old plants (grain-filling stage) of the wild-type and the *pOsLRP5::GUS* transgenic lines for GUS histochemical assays. The samples were incubated in the GUS reaction mixture (1 mM X-gluc, 0.05 mM sodium phosphate buffer, pH 7.0, and 0.1% v/v Triton X-100) under vacuum for 30 min to facilitate infiltration, and further incubated at 37 °C overnight. Green tissues were treated with 70% (v/v) ethanol before observation. Sectioned tissues were imaged under a stereomicroscope coupled to a color CCD camera (Olympus MVX10).

### Southern blot analysis

Genomic DNA (~100 μg) was extracted from leaves of the wild-type and the overexpression lines and digested with *Eco*RI and *Bam*HI overnight at 37 °C, before being separated on 0.8% (w/v) agarose gel and then transferred to a Hybond N^+^ nylon membrane (Amersham). Hybridization was carried out using a digoxigenin-labeled hygromycin-resistant gene as the probe at 65 °C overnight. The blots were washed at 65 °C and examined using a phosphorimager (Typhoon 8600).

### Transient expression of *OsLPR5*

*Agrobacterium tumefaciens*-mediated transient co-expression of *35S::SP*^*OsLPR5*^*::EGFP* and *35S::mCherry::HDEL* in *Nicotiana benthamiana* plants was conducted as previously described by Bürstenbinder *et al.* (2013). Tobacco leaves were collected 3 d after infiltration, and EGFP/mCherry fluorescence was visualized using a confocal laser-scanning microscope (Leica SP8X) on the experimental platform at the College of Resources and Environmental Sciences, Nanjing Agricultural University, China.

### Protein expression, staining, and immunoblotting

The coding sequence of OsLPR5 was cloned into a pGS-21a vector to generate the glutathione-S-transferase (GST) fusion protein. The recombinant constructs and an empty vector (control) were transformed into *E. coli* strain Rosetta (DE3). The *E. coli* cells were induced with 0.5 mM IPTG overnight at 16 °C and collected by centrifugation at 5500 *g* for 10 min at 4 °C. The fusion and control proteins were purified with Ni Sepharose. Equal amounts of purified proteins were separated by 12% (w/v) SDS-PAGE and stained with Coomassie Brilliant Blue. Western blotting was carried out to detect the fused protein. Purified proteins were electrophoresed and transferred to polyvinylidene fluoride membranes using a Trans-Blot Turbo transfer system (Bio-Rad). The membrane was blocked with TBST (150 mM NaCl, 10 mM Tris-Cl, and 0.05% Tween 20, pH 8.0) containing 5% non-fat milk at room temperature for 1 h, and then incubated with primary GST-tag mouse monoclonal antibody (Yeasen Biotech Co., Ltd) for 1 h at room temperature. The membranes were washed with TBST three times for 5 min each and then incubated with the secondary antibody Peroxidase AffiniPure Goat Anti-Mouse IgG (H+L) (Yeasen Biotech Co., Ltd). After washing three times, the signal was visualized with ECL substrate (Millipore) using the ChemiDoc XRS system (Bio-Rad). The dilutions used for the primary and secondary antibodies were 1:10 000 and 1:5000, respectively.

### Ferroxidase assays

Transformed tobacco leaves (0.5–1.0 g) were ground in ice-cold extraction buffer, centrifuged at 8500 *g* for 10 min at 4 °C, and the supernatant was collected as described by Yan *et al.* (2011). For the expression of *OsLPR5* in yeast, its coding sequence (1.913 kb) was expressed in the pYES2 vector under the control of the galactose inducible promoter GAL1 and transformed into *Saccharomyces cerevisiae* strain BY4741. As the control, BY4741 was transformed with an empty vector. Fresh inoculum of the transformed yeast (OD_600_=0.5) was grown for 2 d in 2.5 ml of medium consisting of 5% (w/v) galactose, 0.67% (w/v) yeast nitrogen base without amino acids, and 0.19% (w/v) dropout medium supplements without uracil. Cells were harvested by centrifugation, the pellet was ground with glass beads in an extraction buffer (300 mM NaCl, 100 mM acetate buffer, pH 5.0, 5% w/v glycerol, and a cocktail of protease activity inhibitors), centrifuged at 8500 *g* for 10 min at 4 °C, and the supernatant collected. The supernatants collected from the transformed tobacco leaves and yeast were then used for assaying the ferroxidase activity as described by Müller *et al.* (2015). Briefly, the electron donor ferrous ammonium sulfate [Fe(NH_4_)_2_(SO_4_)_2_.6H_2_O] was used as the substrate and ferrozine [3-(2-pyridyl)-5,6-bis(2-[5-furylsulfonic acid])-1,2,4-triazine], a specific Fe^2+^ chelator, was then added for binding the residual substrate at the end of the reaction. The reaction was initiated by mixing 1050 µl buffer (450 mM Na-acetate, pH 5.8, and 100 mM CuSO_4_), 15 ml of the root protein extract, and 225 ml substrate containing 100 mM CuSO_4_. At regular intervals, 200-ml aliquots were transferred to microtiter plates, and the reaction was quenched by adding 14 ml of ferrozine (18 mM). In the control, the substrate was not added to the reaction mixture. The rate of Fe^2+^ oxidation was calculated from the reduction in the absorbance at 560 nm using a molar absorptivity of ε _560_=25400 M^–1^ cm^–1^ for the Fe^2+^-ferrozine complex, as described by Hoopes and Dean (2004).

### Quantification of soluble Pi, Fe(II), and Fe(III) in the xylem sap

The wild-type and the overexpression lines were grown hydroponically in +P medium for 5 weeks. Plants were then transferred to fresh +P medium. After 6 h, their stems were cut 2 cm above the stem–root junction using a sharp razor blade, and the xylem sap was collected from the cut ends for 1 h using a micropipette, as described by Yokosho *et al.* (2009). The soluble Pi in the sap was quantified as described by Nanamori *et al.* (2004), and Fe(II) and Fe(III) were determined using a QuantiChrom^TM^ Fe assay kit (BioAssay Systems).

### Construction of the expression vectors for mutations in OsLPR5

Using CRISPR-Cas9 gene editing technology to knockout *OsLPR5*, two guide RNAs (gRNA) were designed. These gRNAs were introduced to the intermediate vector pOs-sgRNA via *Bsa*I, and then constructed to the final expression vector pH-Ubi-cas9-7 using Gateway recombination (Invitrogen). The constructs were transformed into *A. tumefaciens* (EHA105) for transformation through mature embryos of the Nipponbare wild-type, as described previously (Upadhyaya *et al.*, 2000; Miao *et al.*, 2013).

### Histological analysis

Roots of the wild-type and the overexpression lines were washed thoroughly with water, then fixed with chloral hydrate solution (8 g chloral hydrate, 1 ml glycerol, and 3 ml water) for 20–40 min. Cells were observed under a differential interference contrast (DIC) microscope (Zeiss Axio Imager). Measurements were made of the development angle and width of the root cells using the ImageJ software.

### RT-PCR and qRT-PCR

Total RNA (~1 µg) was isolated from ground tissues using Trizol reagent (Invitrogen) and treated with RNase-free DNase. First-strand cDNA was synthesized using oligo (dT)-18 primer and Superscript II^TM^ Reverse Transcriptase (Invitrogen). *Actin* was used as an internal control for both RT-PCR and qRT-PCR. The qRT-PCR analysis was performed in triplicate for each sample using SYBR green master mix (Vazyme Biotech Co., Ltd) in a StepOnePlus™ Real-time PCR System (Applied Biosystems). Relative expression levels of the genes were computed using the 2^–ΔΔ*C*T^ method of relative quantification (Livak and Schmittgen, 2001). Gene-specific primers are listed in Supplementary Table S1.

### Quantification of soluble Pi and total Fe

The wild-type and the overexpression lines were grown under +P and –P conditions for 3 weeks, harvested, and washed thoroughly with deionized water. The shoots and roots were separated at the hypocotyl–shoot junction and oven-dried at 80 °C overnight. For quantification of Pi, samples (500 mg) were frozen and then homogenized in 1 ml of 10% (w/v) perchloric acid. The homogenate was diluted 10 times with 5% (w/v) perchloric acid, cooled for 30 min on ice, and then centrifuged at 8500 *g* for 10 min at 4 °C. The supernatant was used for quantification of Pi as described by Nanamori *et al.* (2004). For quantification of the total concentration of Fe, oven-dried samples (200 mg) were homogenized, digested in 5 ml of extra-pure grade HNO_3_ and HClO_4_ (87:13, v/v) at 180 °C, and analysed by inductively coupled plasma-optical emission spectroscopy (ICP-OES) (ThermoFisher Scientific) on the experimental platform at the College of Resources and Environmental Sciences, Nanjing Agricultural University, China.

### ^32^Pi uptake assays

Seedlings (7-d-old) of the wild-type, the *Oslpr5* mutant generated using CRIPSR-Cas9, and the overexpression lines *Ox3* and *Ox6* were grown hydroponically under +P conditions for 7 d. The seedlings were then grown for different time periods (3–24 h for the mutant and 1–7.5 h for *Ox3* and *Ox6*) in 200 ml of +P solution labeled with ^32^Pi (8 μCi of KH_2_^32^PO_4_; Perkin-Elmer). The apoplastic ^32^Pi was then removed by incubating the roots of the seedlings in ice-cold desorption solution (2 mM MES, 0.5 mM CaCl_2_, and 0.1 mM NaH_2_PO_4_, pH 5.5) for 10 min. The seedlings were then blotted dry, and the roots and shoots were harvested separately, with their fresh weights being determined. The tissues were digested in a mixture of HClO_4_ and 30% (v/v) H_2_O_2_ at 28 °C for 8–12 h. A scintillation cocktail (3 ml) was added to the digested tissue and ^32^Pi activity was determined by using a liquid scintillation counter (Tri-Carb 2100, Packard). The ^32^Pi counts in the roots and shoots were used to determine the uptake rates and the shoot/root distribution ratio.

### Statistical analysis

Data were analysed by multiple comparisons of one-way ANOVA using Duncan’s test in the SPSS 20 software (*P*<0.05).

## Results

### OsLPR5 exhibits higher expression in the roots and collar, and is induced during Pi deficiency

**Fig. 1.**
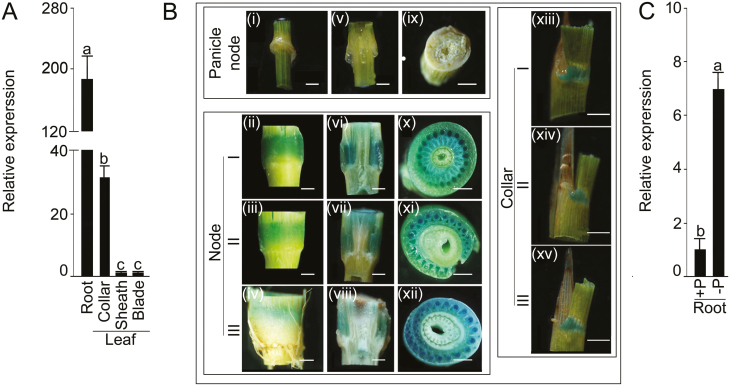
Relative expression levels of *OsLPR5* in different tissues of rice and its tissue specificity. (A, C) Wild-type (WT) plants were grown hydroponically in a nutrient-rich medium for 5 weeks (A), or under Pi-sufficient (+P) or Pi-deficient (–P) conditions for 3 weeks. qRT-PCR was used to determine the relative expression levels of *OsLPR5* in (A) different tissues, and (C) in the roots. *OsRAC1* (LOC_Os03g50885) was used as the internal control. Data are means (±SE), *n*=3. Different letters indicate significant differences between means as determined using ANOVA followed by Duncan’s test (*P*<0.05). (B) Transgenic plants with GUS driven by the *OsLPR5* promoter were grown in soil for 17 weeks (grain-filling stage). GUS activity was then assayed in the panicle nodes (i, v, ix), in node I (ii, vi, x), node II (iii, vii, xi), and node III (iv, viii, xii), and in collar I (xiii), collar II (xiv), and collar III (xv). From left to right, the columns of images show the whole tissues, and their longitudinal and cross-sections. Scale bars are 2 mm.

qRT-PCR analysis was used to determine the expression patterns of OsLRP5, and it was found that expression was far greater in the roots than in the other tissues (Fig. 1A). Expression in the collar was significantly higher than the leaf sheath and blade. To further assess tissue-specific expression, the promoter of *OsLPR5* was fused with the GUS reporter gene and transformed into the Nipponbare wild-type. GUS activity was then assayed histochemically in the tissues of plants grown in soil at the gran-filling stage (Fig. 1B). The activity was observed to be weak in the whole-sections of the panicle nodes and in their longitudinal and transverse sections (Fig. 1Bi, v, ix). In contrast, strong activity was detected in the whole-sections of nodes I–III (Fig. 1Bii–iv). Longitudinal and transverse sections revealed higher GUS activity in the xylem vessels and sieve tubes compared with the other tissues (Fig. 1Bvi–vii, x–xii). High GUS activity was also specific in the collars I–III (Fig. 1Bxiii–xv), suggesting a likely role of *OsLPR5* in the developmental responses of the leaf. Similar patterns of GUS activity driven by the *OsLPR5* promoter were also observed in whole-tissue and cross-sections of nodes I–III, and in collars I–III of two independently generated transgenic lines, #6 and #33 (Supplementary Fig. S1). Notably, GUS activity could not be detected in the roots despite an elevated relative expression level of *OsLPR5* (data not shown). This anomaly could have been due to the fact that the intergenic region (–3384 to –2290 bp) of the GUS reporter gene was not included. To determine the enrichment of *cis*-elements that might influence expression in the roots, this region was annotated by using the PLACE database (http://www.dna.affrc.go.jp/PLACE). This analysis revealed the presence of seven ‘ROOTMOTIFTAPOX1’ (ATATT) motifs in this region, which have been implicated in conferring root-specific expression (Elmayan and Tepfer, 1995).

To determine the effect of Pi deficiency on the relative expression level of *OsLPR5*, wild-type seedlings were grown hydroponically under +P or –P conditions for 3 weeks. Pi deficiency triggered significant induction in the relative expression of *OsLPR5* in the roots (Fig. 1C), consistent with a previous study that reported that Pi deficiency mediated the elevated expression of *OsLPR5* and its homolog *OsLPR3* (Cao *et al.*, 2016).

### Augmented ferroxidase activity in *OsLPR5*-overexpression lines mimics the Pi-deficiency response

Bioinformatic analysis was employed to determine whether OsLPR5 has a potential ferroxidase activity (Supplementary Fig. S2). The MAFFT algorithm (www.ebi.ac.uk/Tools/msa/mafft/) was used for multiple amino-acid sequence alignment of OsLPR5 with AtLPR1/2 and Fet3p from yeast (Supplementary Fig. S2A) and indicated that the Cu sites are organized into three domains, namely a monocluster Cu site (T1), a trinuclear Cu cluster with three amino acid residues (E185, D283, and D409; T2/T3), and a Fe^2+^ substrate-binding site. The T2/T3 site is located in the vicinity of a T1 site, which is critical for binding Fe^2+^ and for electron transfer. The annotation revealed that the amino acid sequences in these regions are largely conserved. In addition, the Phyre2 software (www.sbg.bio.ic.ac.uk/phyre2/) was used to predict the 3D structural model of OsLPR5 (Supplementary Fig. S2B).

**Fig. 2.**
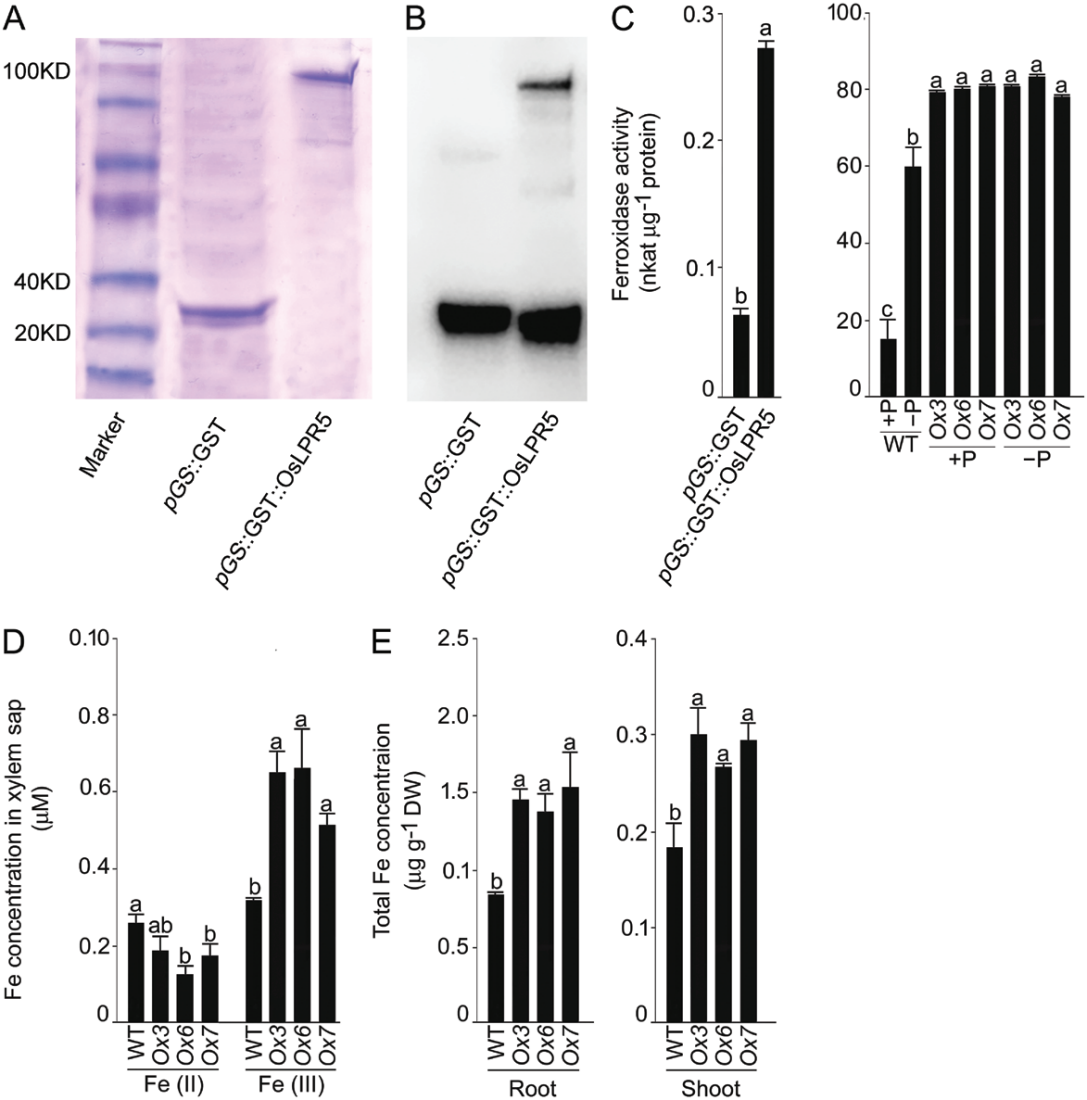
Rice OsLPR5 encodes a ferroxidase. (A, B) Purified *pGS*::GST::OsLPR5 fusion protein was expressed in the *E. coli* strain BL21 (DE3) using the vector inducible by β-D-1-thiogalactopyranoside, and assayed using (A) SDS-PAGE and (B) immunoblotting by raising the antibody against the epitope tag. (C) Ferroxidase activity was assayed in the BL21 strain transformed with *pGS*::GST (control) or with *pGS*::GST::OsLPR5, and in the roots of the rice wild-type (WT) and the *OsLPR5*-overexpression lines *Ox3*, *Ox6*, and *Ox7*. Plants were grown hydroponically under Pi-sufficient (+P) or Pi-deficient (–P) conditions for 3 weeks. (D) Concentrations of Fe(II) and Fe(III) in the xylem sap of the WT and *OsLPR5*-overexpression lines grown hydroponically under +P conditions for 5 weeks. (E) Concentration of total Fe in the roots and shoots of the WT and *OsLPR5*-overexpression lines grown hydroponically under +P conditions for 3 weeks. All data are means (±SE), *n*=3. Different letters indicate significant differences between means as determined using ANOVA followed by Duncan’s test (*P*<0.05).

To investigate whether *OsLPR5* encodes ferroxidase, the OsLPR5 protein was fused with a GST tag, and the purified fusion protein was then heterologously expressed in *E. coli* strain BL21 (DE3) and assayed by SDS-PAGE (Fig. 2A), and immunoblotted using the GST antibody (Fig. 2B). These assays revealed the successful expression of the fusion protein (*pGS*::GST::OsLPR5) compared with the control (*pGS*::GST), and the ferroxidase activity was significantly higher in BL21 expressing the fusion protein compared with the control (Fig. 2C). To determine whether OsLPR5 had ferroxidase activity *in vivo*, transgenic *OsLPR5*-overexpression lines were generated (*Ox3*, *Ox6*, and *Ox7*). RT-PCR and qRT-PCR analyses respectively showed significant increases in transcript abundance and relative expression levels of *OsLPR5* in *Ox3*, *6*, and *7* compared with the wild-type (Supplementary Fig. S3A, B). Southern blotting confirmed the fidelity of the overexpressing lines (Supplementary Fig. S3C).

Assays for ferroxidase in the wild-type indicated that roots of Pi-deprived plants had significantly higher activities (~3-fold) than in Pi-sufficient plants (Fig. 2C). In contrast, the overexpressing lines all had elevated ferroxidase activities (~4-fold) irrespective of the Pi treatment. These results thus revealed the mimicking of the Pi-deficiency response by the transgenic lines overexpressing *OsLPR5*.

In higher plants, Fe is essential for the synthesis of ferredoxins and other redox-related proteins, and in grasses, the chelation-based approach (Strategy II) is used for its acquisition by the roots and subsequent mobilization through the xylem to the aerial parts (Palmer and Guerinot, 2009). Xylem sap is conventionally used for determining the concentrations of different chemical forms of Fe in graminaceous plants (Kawai *et al.*, 2001; Yokosho *et al.*, 2009; Ariga *et al.*, 2014), and hence we took samples of sap to determine the concentrations of Fe(II) and Fe(III). The concentrations of Fe(II) in *Ox6* and *7* were significantly lower (~28–52%) compared with the wild-type, whilst those of Fe(III) in *Ox3*, *6*, and *7* were significantly higher (~1.5–2.0-fold) (Fig. 2D). The total concentration of Fe was also significantly higher in the roots (~64–83%) and shoots (~46–66%) of the *OsLPR5*-overexpression plants compared with the wild-type (Fig. 2E). These results were in agreement with the elevated ferroxidase activity observed in the overexpressors under the different Pi conditions (Fig. 2C).

The growth of the primary root in the *Atlpr1* mutant becomes insensitive to Pi deficiency due to the loss of ferroxidase function (Müller *et al.*, 2015). We therefore hypothesized that overexpressing *OsLPR5* in the *Atlpr1* background would result in complementation of this growth response in Pi-deprived plants. Hence, we transformed the *p35S::OsLPR5* construct into *Atlpr1*, and three independent highly expressing lines were obtained (*OsLPR5/atlpr1 #1, #2,* and *#3*; Supplementary Fig. S4A). Under +P conditions, the primary root growth of *Atlpr1* and the *OsLPR5/atlpr1* lines was comparable with the wild-type (Supplementary Fig. S4B, C) however, under –P conditions, the root length in the wild-type was significantly shorter, which was consistent with previous studies (Williamson *et al.*, 2001; López-Bucio *et al.*, 2002; Jain *et al.*, 2007). Meanwhile, the primary root length of the *Atlpr1* mutant was comparable between the two Pi treatments. The roots of the *OsLPR5/atlpr1* lines under –P conditions were significantly longer than those of the wild-type, but shorter than those of the *Atlpr1* mutant.

### OsLPR5 is localized to both the endoplasmic reticulum and the cell wall

**Fig. 3.**
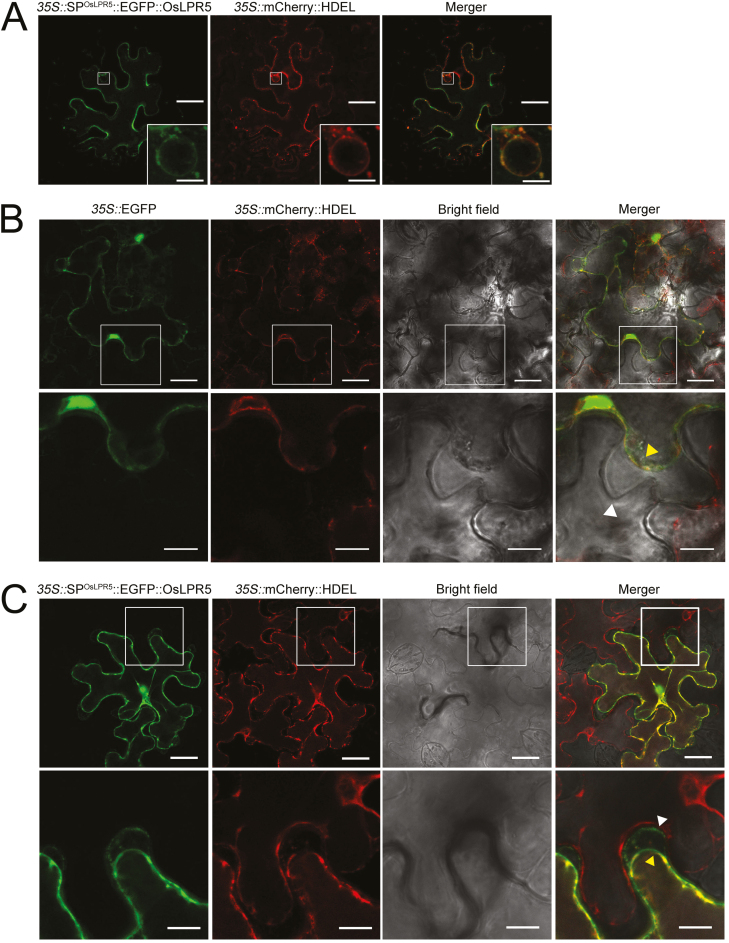
Subcellular localization of OsLPR5 in epidermal leaf cells of transformed *Nicotiana benthamiana* as determined using confocal microscopy. (A) Transient expression of *35S::*SP^OsLPR5^::EGFP::OsLPR5 and *35S::*mCherry::HDEL. Green fluorescent protein (GFP) and mCherry (red) together with the merged images indicate the localization of OsLPR5 to the endoplasmic reticulum. The insets show magnified images of the nucleus. (B, C) After transient expression of (B) *35S::*EGFP and *35S::*mCherry::HDEL, and (C) *35S::*SP^OsLPR5^::EGFP::OsLPR5 and *35S::*mCherry::HDEL, the epidermal leaf cells were plasmolysed by treating with 0.8 M mannitol. The fluorescence and bright-field images demonstrate the localization of OsLPR5 to the cell wall. The areas of the cell walls indicated by the boxes are shown in magnified view in the images below. White arrowheads indicate the cell wall and yellow arrowheads indicate the protoplast membrane. Scale bars are 25 µm in the main images, and 5 µm in the inset in (A) and in the magnified views in (B, C).

The endoplasmic reticulum (ER) acts as a reservoir for proteins destined either for the ER itself or for other organelles (Crofts *et al.*, 2004). A signal peptide at the N-terminal end of the protein is necessary to facilitate its retention in the ER (Gomord *et al.*, 1997; Pagny *et al.*, 1999). Use of SignalP 4.1 (http://www.cbs.dtu.dk/services/SignalP;Petersen *et al.*, 2011) enabled us to identify a putative ER-specific signal peptide in OsLPR5, which was similar to Arabidopsis LPR1 (Ticconi *et al.*, 2009). Ferroxidases typically reside in the extracellular matrix, and LPR1 has been shown to function in the apoplast (Müller *et al.*, 2015). Therefore, to examine the subcellular localization of OsLPR5 as a putative ER-targeted protein, we used mCherry (Nelson *et al.*, 2007). Accordingly, *p35S::SP*^*OsLPR5*^*::EGFP::OsLPR5* and *p35S::mCherry::HDEL* were transiently co-expressed in *N. benthamiana* by *Agro*-infiltration and confocal microscopy revealed the localization of OsLPR5 to the ER (Fig. 3A). To further determine whether OsLPR5 was also targeted to the cell wall, epidermal leaf cells of the transgenic *N. benthamiana* were plasmolysed with 0.8 M mannitol. Although there was distinct plasmolysis in cells expressing *35S::EGFP*, GFP fluorescence could not be detected in the cell wall (Fig. 3B). In contrast, distinct GFP fluorescence was observed in the epidermal cell walls of plants expressing *35S*::*SP*^*OsLPR5*^*::EGFP::OsLPR5* (Fig. 3C).

### Mutation and overexpression of OsLPR5 affects translocation of Pi from the root to shoot

**Fig. 4.**
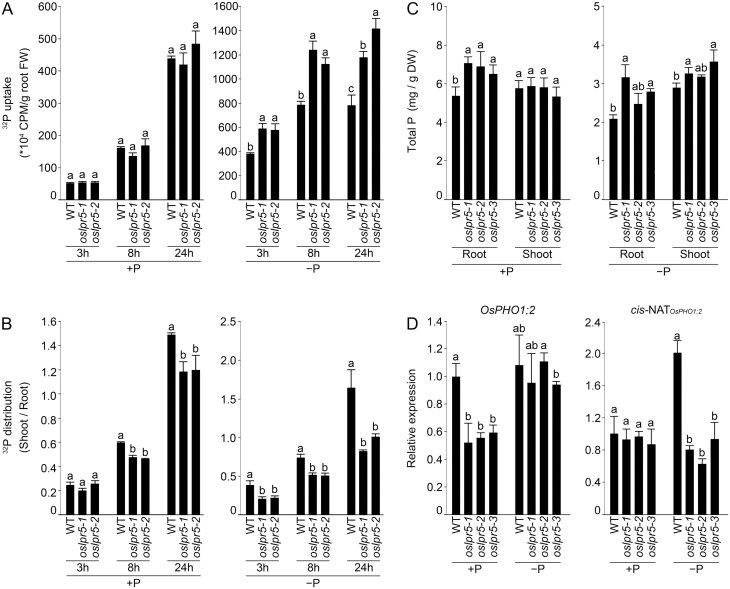
Mutation of *OsLPR5* affects Pi homeostasis in rice. The wild-type (WT) and the *OsLPR5* mutants *oslpr5-1*, *oslpr5-2*, and *oslpr5-3* were grown hydroponically under Pi-sufficient (+P) or Pi-deficient (–P) conditions for 3 weeks. The WT, *oslpr5-1*, and *oslpr5-2* were then fed with ^32^Pi for 10 min. (A) Total ^32^P uptake, (B) shoot/root distribution ratio of ^32^P, and (C) the concentration of total P in the roots and shoots. (D) Relative expression levels of *OsPHO1;2* and *Cis-NAT*_*OsPHO1;2*_ as determined by qRT-PCR in the roots of the WT and *OsLPR5* mutants grown under +P and –P conditions for 3 weeks. *OsRAC1* was used as the internal control. Data are means (±SE), *n*=3. Different letters indicate significant differences between means as determined using ANOVA followed by Duncan’s test (*P*<0.05).

To determine the role of *OsLPR5* in the maintenance of Pi homeostasis in rice, CRISPR/Cas9 gene editing was used to generate homozygous *OsLPR5* knockout mutants, of which three independent lines (*Oslpr5-1*, *5-2*, and *5-3*) were selected for further study (Supplementary Fig. S5). Since *OsLPR5* was induced during Pi deficiency (Fig. 1B), the effects of the mutation on Pi homeostasis were investigated (Fig. 4). The wild-type and mutants were grown hydroponically under +P and –P conditions for 2 weeks, and were then treated with ^32^Pi for different time periods. Under +P conditions, the ^32^Pi uptake rate was consistently comparable between the wild-type and the mutants (Fig. 4A); in contrast, under –P conditions the uptake was significantly higher in the mutants. Meanwhile, the shoot/root distribution ratio of ^32^P distribution was significantly lower in the mutants compared with the wild-type under both P conditions and at all the time-points (Fig. 4B). In addition, there was a significant increase in the concentration of total P in the mutants compared with the wild-type (+P roots for *oslpr5-1*, *5-2*, and *5-3*, and –P roots and shoots for *oslpr5-1* and *5-3*Fig. 4C). These data suggested an inhibitory effect of the mutation in *OsLPR5* on the translocation of Pi from the root to shoot. No significant difference could be detected in the concentration of total Fe between the wild-type and the mutants (data not shown). Compared with the wild-type, a significant increase in ^32^Pi uptake by the overexpressor lines *Ox3* and *Ox6*) could be observed by as early as 3 h under +P conditions, and this trend continued through to the end of the experiment at 7.5 h (Supplementary Fig. S6A). In contrast, under –P conditions there were no differences between the wild-type and the overexpressing lines. The concentration of Pi in the xylem sap was significantly higher in the two overexpressor lines than in the wild-type under both *+*P and –P conditions (Supplementary Fig. S6B). Irrespective of the Pi conditions, there were no significant differences in the shoot/root distribution ratio of ^32^Pi between the wild-type and the overexpressor lines (Supplementary Fig. S6C). However, the shoot Pi concentration in the overexpressor lines was significantly higher under both +P and –P conditions compared with the wild-type (Supplementary Fig. S6D).

*OsPHO1;2* is involved in Pi loading into the xylem and thus facilitates its mobilization from the roots to shoots in rice (Secco *et al.*, 2010). Natural antisense transcripts (NATs) are long RNAs comprising sequences that are complementary to sense mRNAs, and they are categorized into *cis* and *trans* forms (Lapidot and Pilpel, 2006). *cis*-NATs are prevalent in plants and are often associated with the down-regulation of their associated sense genes (Swiezewski *et al.*, 2007; Held *et al.*, 2008; Ron *et al.*, 2010). Since the Pi concentration in the shoots were consistently higher in the overexpressor lines (SupplementaryFig. S6D), we examined the effects of mutation in *OsLPR5* on the relative expression levels of *OsPHO1;2* and *cis*-NAT_*OsPHO1;2*_. Under +P conditions the relative expression level of *OsPHO1;2* in the mutants was significantly lower compared with the wild-type (Fig. 4D), whilst it was comparable under –P conditions. In contrast, Pi deficiency triggered a significant reduction in the relative expression of *cis*-*NAT*_*OsPHO1;2*_ in the mutants compared with the wild-type.

### Mutation and/or overexpression of OsLPR5 affects root development and other morphological traits

**Fig. 5.**
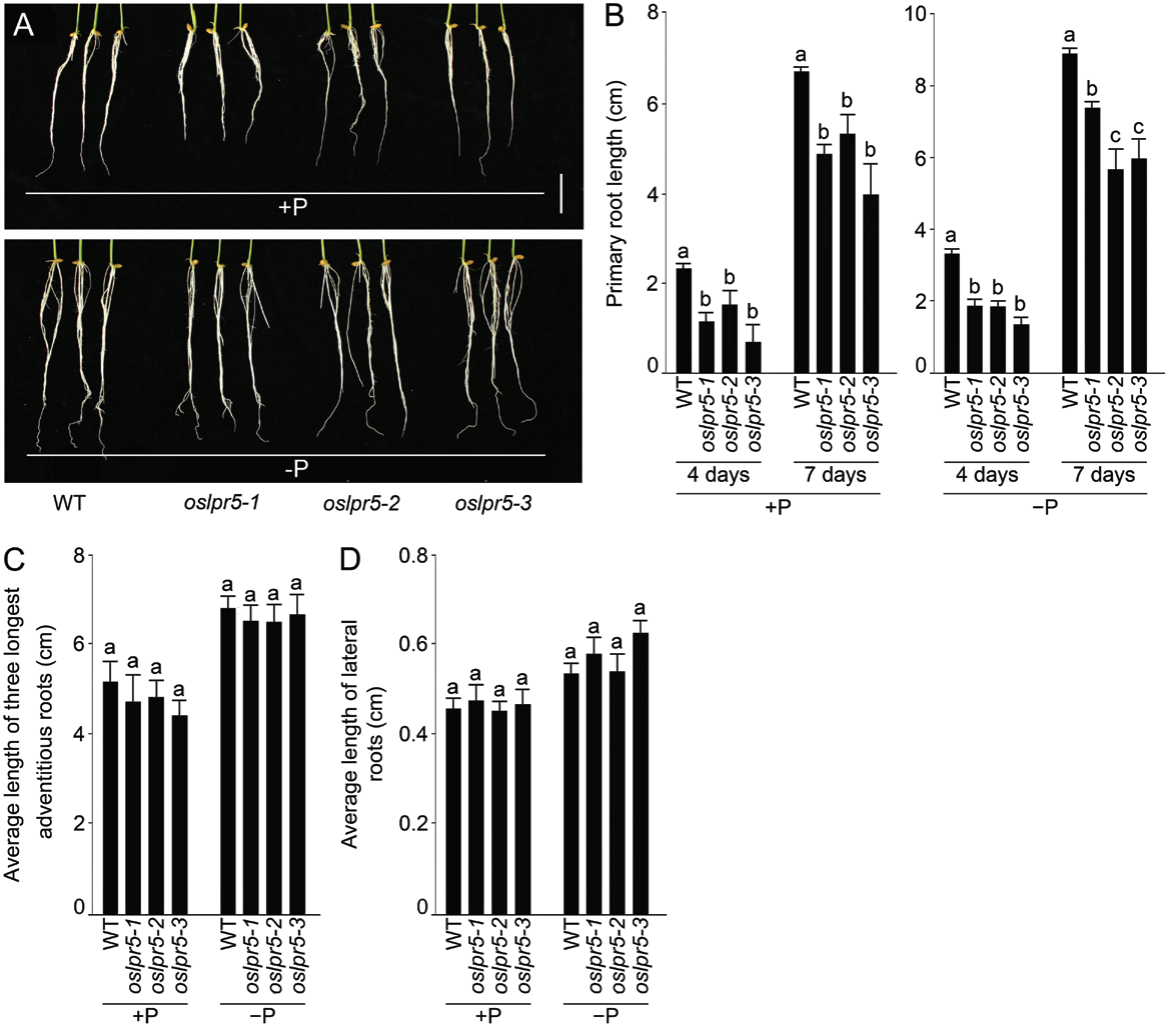
Effects of mutation of *OsLPR5* on the growth of roots in rice grown under different Pi conditions. Seedlings (4 d old) of the wild-type (WT) and the *OsLPR5* mutants *oslpr5-1*, *oslpr5-2*, and *oslpr5-3* were grown hydroponically under Pi-sufficient (+P) or Pi-deficient (–P) conditions. (A) Images of the roots after 7 d. (B) Length of the primary root after 4 d and 7 d, (C) mean length of the three longest adventitious roots after 7 d, and (D) length of lateral roots after 7 d. Data are means (±SE), *n*=6. Different letters indicate significant differences between means as determined using ANOVA followed by Duncan’s test (*P*<0.05).

The root system exhibits extensive developmental plasticity in response to various environmental cues (Malamy and Ryan, 2001). In rice, the primary and lateral roots constitute a fibrous network and the latter contribute towards branching, which is crucial for anchorage and the acquisition of nutrients (Hochholdinger and Zimmermann, 2008; Atkinson *et al.*, 2014). We therefore examined the effects of mutation of *OsLPR5* on different root traits during growth in a hydroponic system under different Pi conditions. The wild-type and the *oslpr5-1*, *oslpr5-2*, and *oslpr5-3* mutants were grown hydroponically under +P or –P conditions for 4 d or 7 d. There was a significant reduction in the primary root length of the mutants compared with the wild-type after 4 d in both the +P and –P treatments (Fig. 5A, B). However, irrespective of the Pi conditions, there were no effects of the on the mean length of the three longest adventitious roots (Fig. 5C), or on the mean length of the lateral roots (Fig. 5D).

**Fig. 6.**
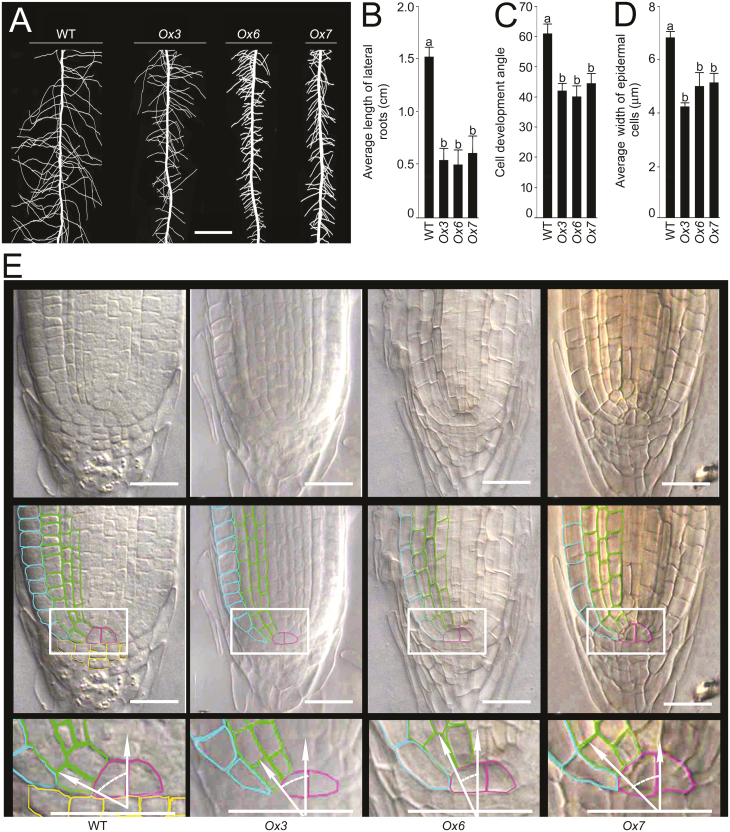
Overexpression of *OsLPR5* reduces lateral root elongation in rice. Seedlings (4 d old) of the wild-type (WT) and the *OsLPR5*-overexpression lines *Ox3*, *Ox6*, and *Ox7* were grown hydroponically in Pi-sufficient medium for 7 d. (A) Images showing lateral root development on the primary roots. The scale bar is 1 cm. (B) Length of lateral roots, (C) cell development angle (see below), and (D) width of epidermal cells. Data are means (±SE), *n*=6. Different letters indicate significant differences between means as determined using ANOVA followed by Duncan’s test (*P*<0.05). (E) Sections of the lateral root tip (top row) together with schematic representations (middle row). Blue indicates the epidermis, green is the cortex, purple is the quiescent center, and yellow indicates stem cells. Magnified views of the areas within the boxes are shown in the bottom row of images, and illustrate the cell development angle, i.e. the angle between the axis of the direction of growth of the root and the lower cortical cells. Scale bars are 20 µm.

To further examine whether the overexpression of *OsLPR5* exerted any influence on the developmental responses of different root traits, the wild-type and the overexpressing lines were grown hydroponically in +P medium for 7 d. The laterals on the primary roots were then spread gently to reveal their architectural details, and different traits were quantified by using the ImageJ software (https://imagej.nih.gov/ij/;Collins, 2007). The primary root length of the overexpressing lines was comparable with that of the wild-type (data not shown). However, an adverse effect on lateral root development was observed in the overexpressing lines (Fig. 6A) in the form of a significant reduction in the mean length (Fig. 6B). Significant reductions were also observed in the cell development angle and in the mean width of the epidermal cells of the overexpressing lines compared with the wild-type (Fig. 6C, D). However, the length of epidermal cells of the lateral roots was comparable between the wild-type and the overexpressing lines (data not shown). To determine whether overexpression of *OsLPR5* caused any perturbations in the spatial arrangements of the quiescent center and the surrounding stem, cortical, and epidermal cells of the lateral root tip, we observed sections under a stereomicroscope, and found that there was a significant reduction in the width of the cells in the overexpressing lines compared with the wild-type (Fig. 6E), and also in the angle between the axis of growth and the lower cortical cells.

**Fig. 7.**
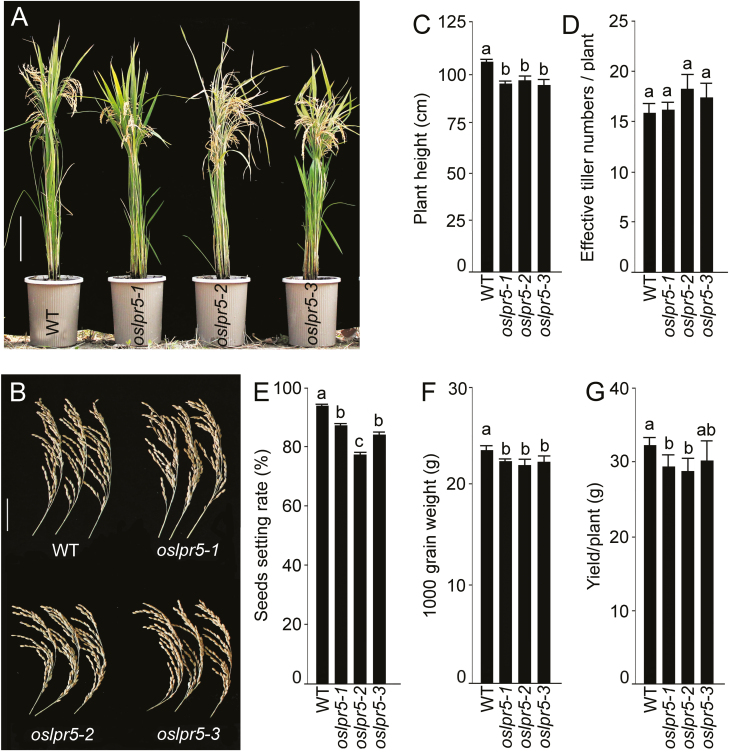
Effects of mutation of *OsLPR5* on various morphological traits in rice. The wild-type (WT) and *OsLPR5* mutants *oslpr5-1*, *oslpr5-2*, and *oslpr5-3* were grown in soil in Pi-sufficient conditions for 17 weeks (grain-filling stage). (A, B) Images of the plants and panicles. Scale bars are 20 cm (A) and 5 cm (B). (C) Plant height, (D) effective tiller number per plant, (E) seed-setting rate, (F) 1000-grain weight, and (G) grain yield per plant. Data are means (±SE), *n*=6. Different letters indicate significant differences between means as determined using ANOVA followed by Duncan’s test (*P*<0.05).

Finally, the effects of O*sLPR5* mutation on different morphological traits were examined at the grain-filling stage in plants grown in soil supplemented with Pi fertilizer. The *Oslpr5* mutants appeared relatively stunted compared with the wild-type (Fig. 7A) due to a significant reduction in their height (Fig. 7C). There were no observable effects of the mutation in the panicles (Fig. 7B) or on the effective number of tillers per plant (Fig. 7D); However, there were significant reductions in the seed-setting rate (Fig. 7E), the 1000-grain weight (Fig. 7F), and in the grain yield per plant (Fig. 7G).

## Discussion

### *OsLPR5* is expressed in the roots and the collar, and is responsive to Pi deficiency

The expression of *OsLPR5* was significantly higher in the roots compared with the other tissues (Fig. 1A). A previous study also reported higher expression levels of *OsLPR1*, *3*, *4*, and *5* in the roots, with the notable exception of *OsLPR2* (Cao *et al.*, 2016), suggesting a potential functional overlap amongst the members of the *OsLPR* family. Strong GUS activity driven by the *OsLPR5* promoter was observed in the xylem vessels and sieve tubes of the nodes I–III (Fig. 1B). Since nodes are junctions of vasculatures that connect leaves, stems, and panicles, and act as hubs in redirecting essential mineral elements taken up by the roots to different organs (Yamaji and Ma, 2014), a role of *OsLPR5* in the mobilization of minerals can thus be envisaged. Although the expression of *OsLPR5* in the collar was relatively lower than in the roots, it was significantly higher compared with the leaf sheath and blade (Fig. 1A). In addition, the GUS activity was high in collars I–III (Fig.1B). Cell proliferation in the leaf collar (also referred to as the lamina joint) is closely related to leaf erectness in rice (Sun *et al.*, 2015; Ruan *et al.*, 2018). Ruan *et al.* (2018) found that *Regulator of Leaf Inclination1* (*RLI1*) shows strong expression in the collar and positively regulates leaf inclination in rice by influencing cell elongation in the lamina joint. However, during Pi deficiency, the proteins SPX1 and SPX2 are induced and interact with RLI1, thus repressing its activity. Hence, RLI1 and SPX form a module to regulate leaf inclination in response to Pi availability in rice. The expression of *OsLPR5* in the collar does suggest it has a potential role in the regulation mechanism for leaf inclination; however, further studies will be required to examine this more closely.

Overall, the spatial expression pattern of *OsLPR5* was consistent with the findings of Cao *et al.* (2016), who determined expression of *OsLPR* homologs not only in the roots, but also in leaf blades, leaf sheaths, and other tissues. Variable expression levels in different tissues of several functionally diverse genes have often been correlated with their diverse roles, for example *OsPT2* and *OsPT4* (encoding Pi transporters), *OsWRKY74* (encoding a transcription factor), and *OsLTN1* (an ortholog of Arabidopsis *PHO2*) (Ai *et al.*, 2009; Hu *et al.*, 2011; Zhang *et al.*, 2015; Dai *et al.*, 2016). Multiple tissue-specific roles of *OsLPR5* may thus be assumed. Pi deficiency in Arabidopsis does not exert any significant effect on the transcription of *AtLPR1* (Svistoonoff *et al.*, 2007; Müller *et al.*, 2015). In contrast, in our study a significant increase in the relative expression level of *OsLPR5* in Pi-deprived roots was observed (Fig. 1C) and this was consistent with an earlier study that reported elevated expression levels of *OsLPR5* and its homolog *OsLPR3* in response to Pi deficiency (Cao *et al.*, 2016).

### Overexpression of *OsLPR5* triggers elevated ferroxidase activity and mimics the Pi deficiency response

Several studies in Arabidopsis have demonstrated a significant role of the antagonistic interaction between Pi and Fe in maintaining nutrient homeostasis (Misson *et al.*, 2005; Hirsch *et al.*, 2006; Ward *et al.*, 2008; Bournier *et al.*, 2013; Müller *et al.*, 2015; Dong *et al.*, 2017; Gutiérrez-Alanís *et al.*, 2017; Wang *et al.*, 2019). *AtLPR1* plays a pivotal role in Pi deficiency-mediated developmental responses of the root system architecture in a Fe-dependent manner (Müller *et al.*, 2015). In rice, physiological and transcriptome analyses of the interaction between Pi and Fe has shown that the presence of the former can affect the availability of the latter, and can consequently influence the regulation of Fe-responsive genes (Zheng *et al.*, 2009). Secco *et al.* (2013) used next-generation RNA-seq in conjunction with a comprehensive time-course experiment in order to decipher the spatiotemporal molecular responses of the rice transcriptome and small RNA-ome in response to Pi deprivation and resupply. As well as identifying several key potential regulators of Pi homeostasis, they also found up-regulation of Fe-responsive genes in the roots (two putative vacuolar iron transporters) and shoots (two ferritins and one Fe transporter) in response to short-term (6-h) Pi deprivation.

Multicopper oxidases (MCOs) are a varied group of enzymes that couple the oxidation of an array of substrates to the reduction of dioxygen, and include members such as Fet3p, ascorbate oxidase, laccases, and ceruloplasmin (Solomon *et al.*, 1996). *LPR1/2* and *OsLPR5* encode MCOs in Arabidopsis and rice, respectively (Svistoonoff *et al.*, 2007; Cao *et al.*, 2016). In *Saccharomyces cerevisiae*, the high-affinity Fe-uptake complex in the plasma membrane consists of Fet3p, which is involved in the ferroxidase reaction that catalyses the oxidation of Fe(II) to Fe(III) using O_2_ as a substrate (Van Ho *et al.*, 2002; Singh *et al.*, 2006). Multiple amino-acid sequence alignment of OsLPR5 with AtLPR1/2 and Fet3p revealed the presence of three conserved Cu sites (T1, T2/T3, and a Fe^2+^ substrate binding site) that are crucial for the ferroxidase activity (Supplementary Fig. S2A), and the predicted 3D structural model of OsLPR5 (Supplementary Fig. S2B) was also comparable with those of AtLPR1/2 and Fet3p (Müller *et al.*, 2015). To demonstrate empirically that *OsLPR5* encodes a ferroxidase, we heterologously expressed the purified *pGS*::GST::OsLPR5 fusion protein (Fig. 2A, B). The ferroxidase activity was significantly higher in the *E. coli* strain BL21 expressing the fusion protein compared with the control (*pGS*::GST) (Fig. 2C). In addition, Pi deficiency triggered a significant increase in the ferroxidase activity in the roots of the wild-type (Fig. 2C). Interestingly, *OsLPR5*-overexpressing lines grown hydroponically for 3 weeks under +P and –P conditions both had significantly higher ferroxidase activity compared with the Pi-deprived wild-type. In Arabidopsis, the concentration of Fe has been shown to increase during Pi deficiency (Misson *et al.*, 2005). Consistent with this, the concentrations of Fe(III) in the xylem sap and total Fe in the roots and shoots of *OsLPR5*-overexpressing lines were significantly higher than those of the wild-type (Fig. 2D, E). Our results thus demonstrated that the overexpression of *OsLPR*5 mimicked the Pi-deficiency response. Furthermore, the partial complementation of the response of the primary root of the *Atlpr1* mutant of Arabidopsis to Pi deficiency that resulted from overexpressing *OsLPR*5 in the *Atlpr1* background (Supplementary Fig. S4) demonstrated the ferroxidase activity of OsLPR5. Our results thus suggest a pivotal role of *OsLPR5* in mediating cross-talk between Pi and Fe in rice. In addition, transient expression in *N. benthamiana* revealed the subcellular localization of OsLPR5 to both the ER and the cell wall (Fig. 3). These results are consistent with earlier studies on Arabidopsis LPR1 that have demonstrated its targeting to the ER (Ticconi *et al.*, 2009), and to the cell wall, which is crucial for the response of the primary root to local Pi availability (Müller *et al.*, 2015).

### *OsLPR5* plays a role in the maintenance of Pi homeostasis and in developmental responses

To determine whether *OsLPR5* plays any role in the maintenance of Pi homeostasis in rice, knockout mutants were generated (SupplementaryFig. S5). ^32^Pi uptake rates at different time-points were significantly higher in the mutants compared with the wild-type under –P conditions (Fig. 4A), whilst the shoot/root distribution ratio of ^32^P was significantly lower in the mutants under both +P and –P conditions (Fig. 4B). The mutation in *OsLPR5* also resulted in a higher concentration of total P in the roots under +P conditions and in both the roots and shoots under –P conditions compared with the wild-type (Fig. 4C). In the *OsLPR5*-overexpressing lines there was a significant increase in ^32^Pi uptake under +P conditions, and higher concentrations of Pi in the xylem sap and in the shoots Pi under both +P and –P conditions compared with the wild-type (Supplementary Fig. S6). Overall, these results suggest a role for *OsLPR5* in the translocation of Pi from the root to shoot. This was further corroborated by reduced expression of *cis*-*NAT*_*OsPHO1;2*_ in the mutants compared with the wild-type in response to Pi deficiency (Fig. 4D). *cis*-*NAT*_*OsPHO1;2*_ is essential for maintaining OsPHO1;2 under Pi-deficient conditions (Jabnoune *et al.*, 2013). Thus, the reduced expression of *cis*-*NAT*_*OsPHO1;2*_ in the *OsLPR5* mutants would have caused lower expression of the OsPHO1;2 protein, thereby resulting in reduced translocation of Pi from the root to shoot. However, it is not clear how *OsLPR5* would have negatively regulated *cis*-*NAT*_*OsPHO1;2*_ and hence this merits further investigation.

*OsLPR5* also played an important role in the developmental responses of ontogentically distinct root traits. For example, the primary root of the mutants were significantly shorter under both +P and –P conditions compared with the wild-type (Fig. 5A, B), thus highlighting the role of *OsLPR5* in the developmental response of embryonically developed primary roots through both Pi-dependent and Pi-independent pathways. In contrast, the *OsLPR5*-overexpression lines exhibited defects in lateral root development compared with the wild-type (Fig. 6A), as indicated by significant reductions in the mean length of lateral roots (Fig. 6B), in the cell development angle (Fig. 6C), and in the mean width of the epidermal cells (Fig. 6D). Sections of the lateral roots of the *OsLPR5*-overexpression lines revealed defects in the spatial arrangements of the quiescent center and the surrounding stem, cortical, and epidermal cells (Fig. 6E). We assume that these defects in the root tips of the overexpressing lines could have contributed to the reduced lateral root growth. *LPR1* in Arabidopsis has been implicated in adjusting the meristem activity of the primary root upon sensing changes in external Pi (Svistoonoff *et al.*, 2007; Ticconi *et al.*, 2009; Müller *et al.*, 2015); however, *LPR1* has not been reported to exert any apparent influence on the developmental responses of the lateral roots. Our study has thus highlighted a functional divergence between *LPR1* in Arabidopsis and *OsLPR5* in rice on their influence on the ontogenetically distinct embryonically developed primary roots and the post-embryonically developed lateral roots. In Arabidopsis, it has been shown that the blue-light effect in Petri dishes is the actual cause of the Fe-dependent Pi deficiency-mediated inhibition of primary root growth (Zheng *et al.*, 2019). For this reason, in all our experiments seedlings were grown in a hydroponic system with the container painted black to ensure complete exclusion of light from the growing root system. The adverse effects of the mutation in *OsLPR5* were also evident in various morphological traits of plants grown to maturity in soil supplemented with Pi fertilizer. The height of the mutants was significantly reduced compared with the wild-type (Fig. 7A, C), and they also exhibited significant reductions in seed-setting rate (Fig. 7E), 1000-grain weight (Fig. 7F), and grain yield per plant (Fig. 7G). These results therefore suggest that *OsLPR5* has a broad-spectrum influence on the agronomic traits of rice.

### Conclusions

Our study provides evidence that OsLPR5 is a ferroxidase, and that it plays a broad-spectrum role in influencing a subset of the traits that govern Pi homeostasis. It also influences the developmental responses of different root traits and agronomic traits. We have previously shown that *OsPDR2* (homolog of *AtPDR2*) plays important roles in the development responses and maintenance of Pi homeostasis in rice (Cao *et al.*, 2020). In future studies it would be interesting to determine whether *OsLPR5* and *OsPDR2* interact genetically to regulate Pi and/or Fe homeostasis and/or developmental responses under different Pi and Fe regimes.

## Supplementary data

Supplementary data are available at *JXB* online.

Fig. S1. Histochemical analysis of GUS activity driven by *OsLPR5* promoter.

Fig. S2. Sequence alignment of OsLPR5 with Fet3p, AtLPR1, and AtLPR2, and 3D structural model of OsLPR5.

Fig. S3. Expression analysis and Southern blotting of the *OsLPR5*-overexpression lines.

Fig. S4. Complementation of the *Atlpr1* phenotype by the overexpression of *OsLPR5*.

Fig. S5. Identification of the *Oslpr5* mutant lines.

Fig.S6. Effects of *OsLPR5* overexpression on Pi homeostasis.

Table S1. The primers used for promoter–GUS fusion, overexpression, RT-PCR, and qRT-PCR.

Table S2. The primers used for the generation and identification of the mutation of *OsLPR5* by CRISPR/Cas9.

eraa211_suppl_Supplementary_Material
