# Supplementary material for: The ferroxidase LPR5 functions in the maintenance of phosphate homeostasis and is required for normal growth and development of rice
Source: J Exp Bot. 2020 Jul 3;71(16):4828–42. doi: 10.1093/jxb/eraa211 (PMC7475252; doi:10.1093/jxb/eraa211)
Supplement: eraa211_suppl_Supplementary_Material [file eraa211_suppl_supplementary_material.pdf]

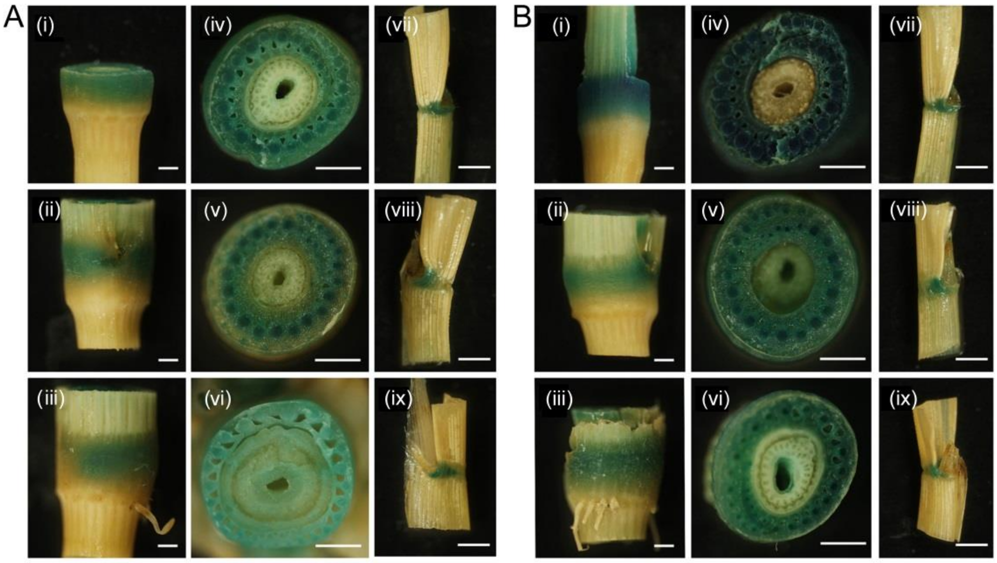

Fig. S1. Histochemical analysis of the GUS activity driven by *OsLPR5* promoter. (A, B) Two independently generated rice transgenic lines (A) #6 and (B) #33 were grown in the pots for 17 weeks (grain-filling stage). The GUS activity driven by *OsLPR5* promoter was assayed in the (i-iii) whole tissue and (iv-vi) cross-sections of the nodes Ⅰ-Ⅲ, and (vii-ix) collars Ⅰ-Ⅲ. Bar = 2 mm.

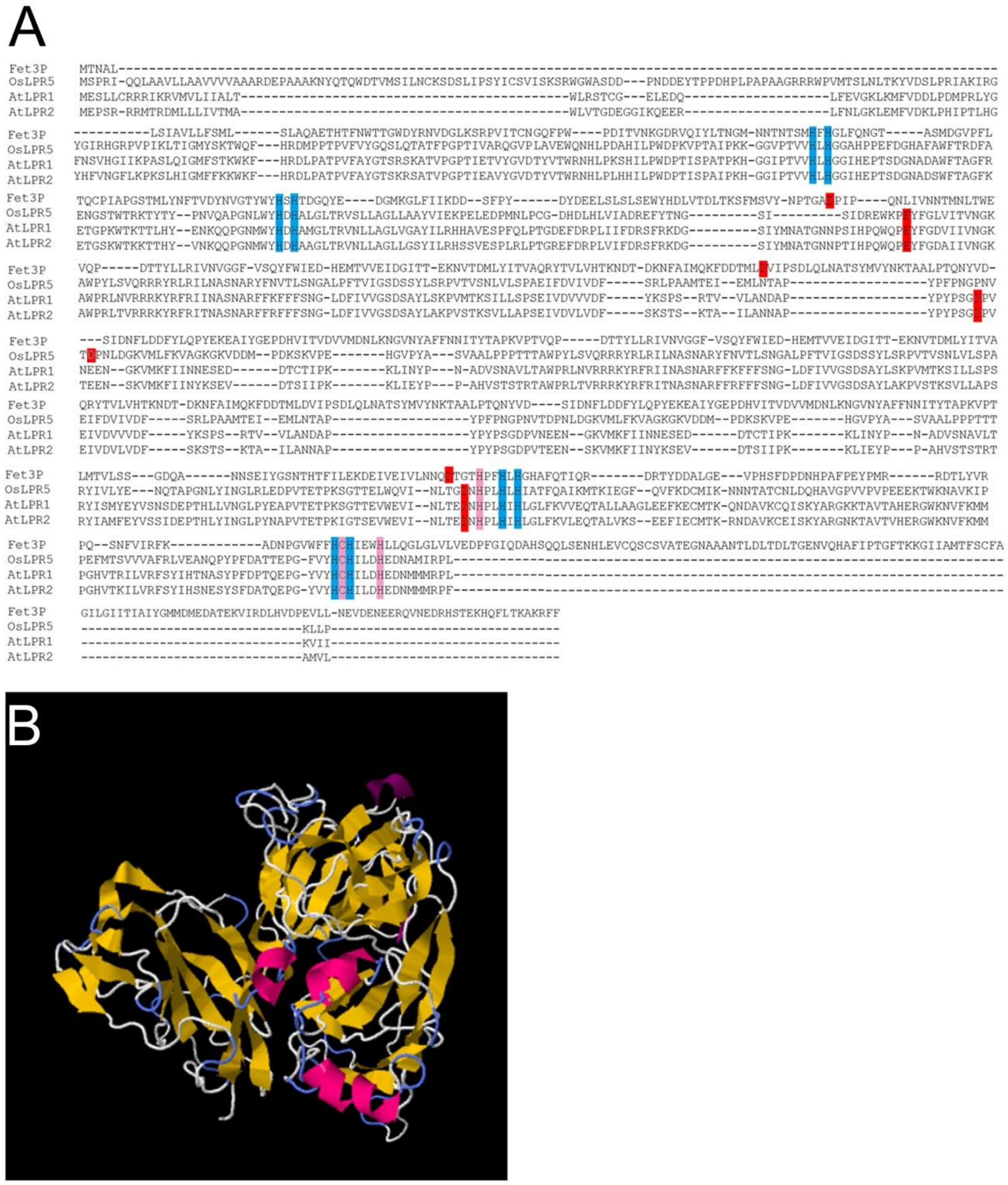

Fig. S2. Bioinformatic analysis for OsLPR5 ferroxidase activity.

(A) Multiple amino acid sequence alignment of Fet3p, AtLPR1/2, and OsLPR5 using the MAFFT algorithm. Sequences highlighted in pink, blue, and red indicate T1, T2/T3, and Fe^{2+} substrate binding sites, respectively. (B) 3D structural modeling of OsLPR5 by Phyre2 software.

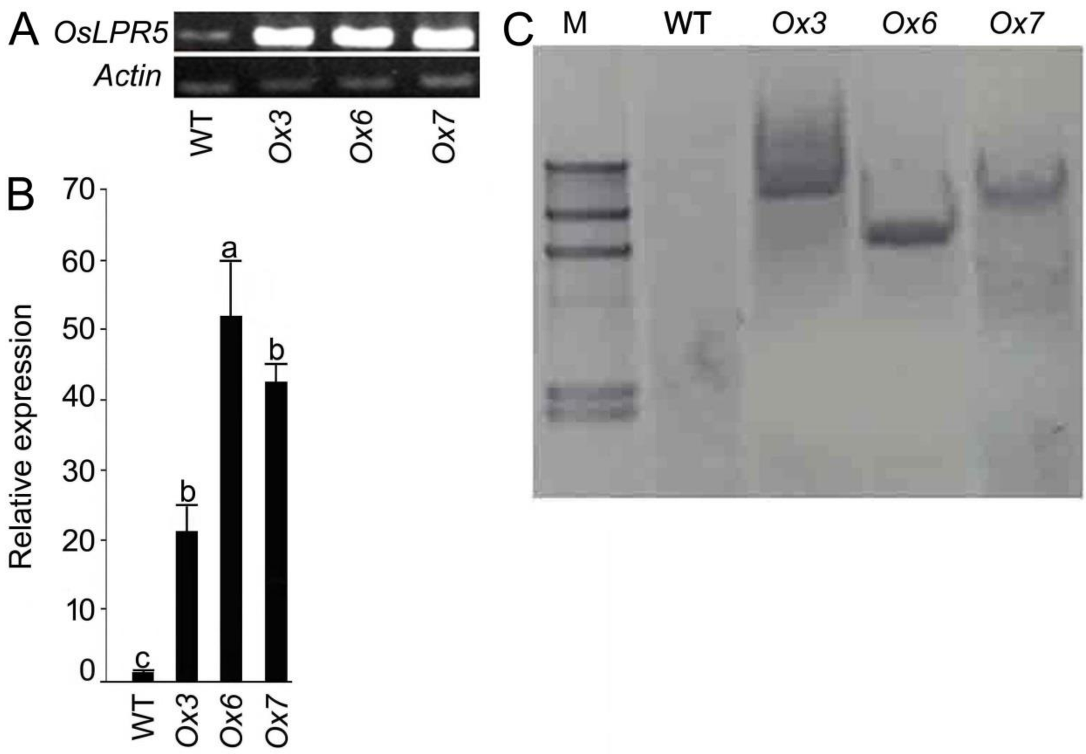

Fig. S3. Expression and Southern blot analyses of *OsLPR5* overexpressor lines. The WT and overexpressor lines (*Ox3*, *Ox6*, and *Ox7*) were grown hydroponically in +P medium for 2 weeks. Roots were harvested for the expression analysis of *OsLPR5*. (A) Semi-quantitative RT-PCR analysis. (B) qRT-PCR analysis. *OsRAC1* was used as an internal control. Values are means ± SE (n = 3) and different letters on the histograms indicate that the values differ significantly ($P < 0.05$). (C) Genomic DNA of the WT and overexpressor lines was restricted with *Hin*dⅢ and *Eco*RⅠ, and the hygromycin gene was used as a probe for Southern blot analysis.

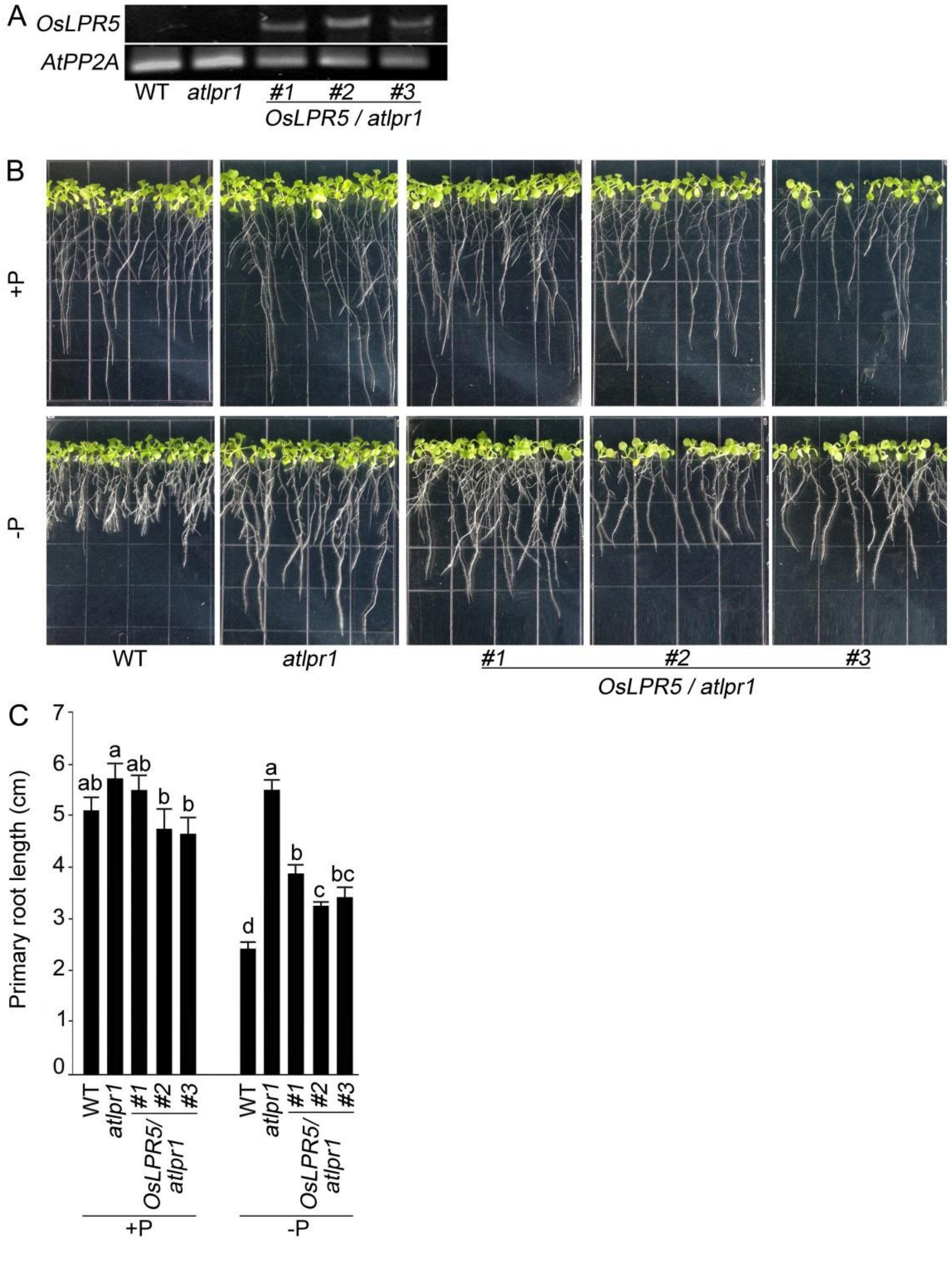

Fig. S4. Complementation of *atlpr1* phenotype by the overexpression of *OsLPR5*. (A) mRNA was isolated from the seedlings of the WT, *atlpr1*, and three independently generated transgenic lines of *atlpr1* complemented with *p35S::OsLPR5* (*OsLPR5*/ *atlpr1#1,#2,* and *#3*), and the transcript abundance of *OsLPR5* was assayed by semi-quantitative RT-PCR. *AtPP2A* (At1g69960) was used as the reference gene. (B) The WT, *atlpr1*, and *OsLPR5*/ *atlpr1#1,#2, #3* were grown on vertically oriented +P and -P agar Petri plates for 7 d and photos were taken to document their phenotypes. (C) Data are presented for the primary root length of the WT, *atlpr1*, and *OsLPR5*/*atlpr1#1*, *#2*, *#3* seedlings grown under +P and -P conditions. Values are means ± SE (n = 8) and different letters on the histograms indicate that the values differ significantly ($P < 0.05$).

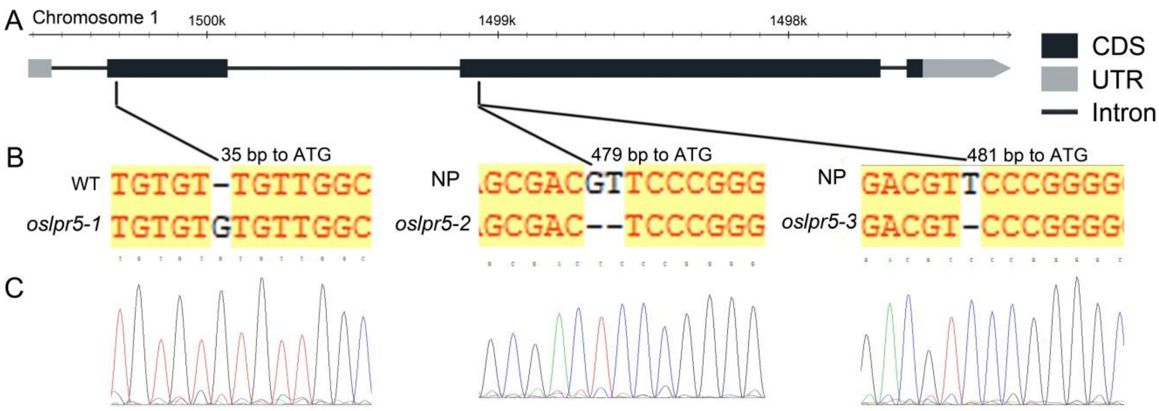

Fig. S5. Identification of *oslpr5* mutant lines.

(A) The gene structure of *OsLPR5*. The coding sequences (CDS), the untranslated regions (UTR) and the introns are indicated by black boxes, grey boxes, and black bars, respectively. (B) The mutation sites of three independent lines and the comparative nucleotide sequences of the WT, and *OsLPR5* mutants. (C) The peak maps of *OsLPR5*mutants sequences.

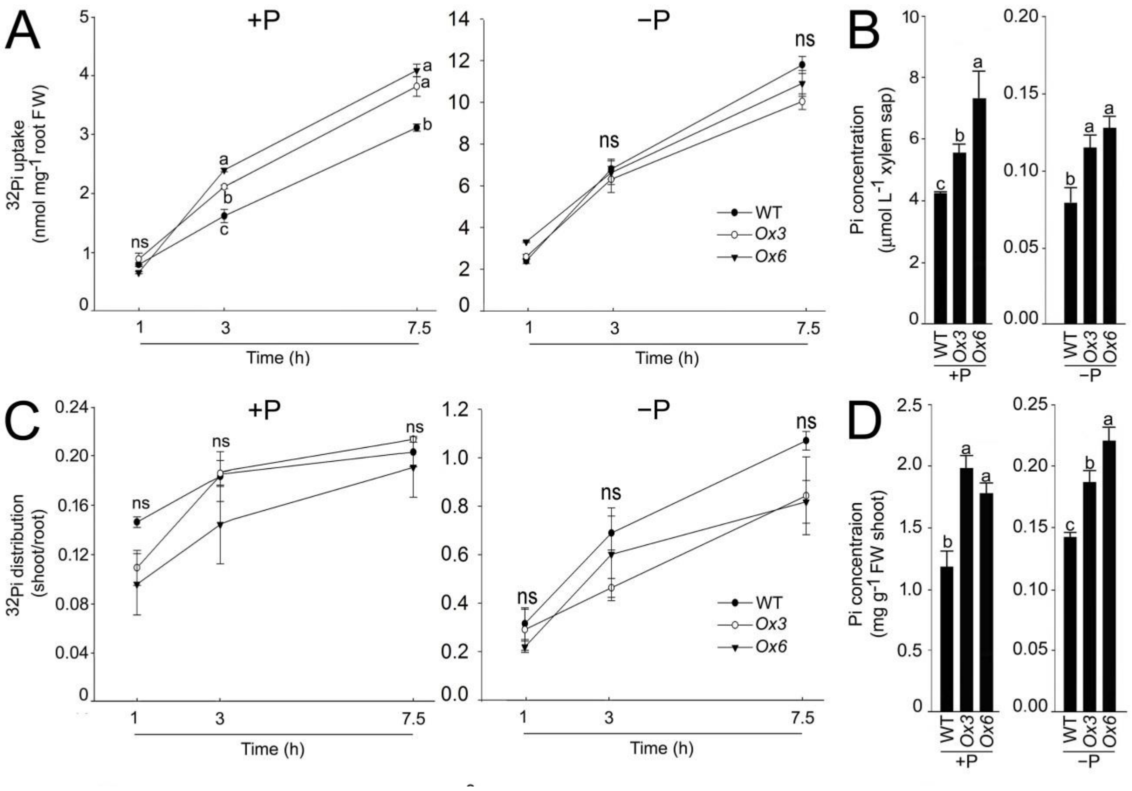

Fig. S6. *OsLPR5* overexpression affects Pi homeostasis.

The WT and overexpressor lines (*Ox3* and *Ox6*) were grown hydroponically under +P and -P conditions for (A and C) 1 week, (D) 3 weeks, and (B) 5 weeks. (A-D) Data are presented for (A) ^{32}Pi uptake, (B) Pi concentration in the xylem sap, (C) ratio of ^{32}Pi distribution in the shoot/root, and (D) Pi concentration in the shoot. Values are means ± SE (n = 3) and different letters on the histograms indicate that the values differ significantly (P < 0.05).

Supplemental Table S1. The primers used for promoter-GUS fusion, overexpression, RT-PCR and qRT-PCR

Gene		Sequences
*OsLPR5*promoter*::GUS*	F(5'-3')	AGCTGGTACCAGCATACTCAAGGACAAC
	R(5'-3')	CGCAGGATCCTGATTGCATTCTAAGTTT
Ubiquitin::OsLPR5	F(5'-3')	GCCGGATCCCCTTTAATTCATTATCCTTTGC
	R(5'-3')	GCACTAGTGATCAGCACTACGTTTCTTGTT
OsActin-RT- PCR	F(5'-3')	CCTCGTCTCGACCTTGCTGGG
	R(5'-3')	GAGAACAAGCAGGAGGACGGC
OsLPR5-RT- PCR	F(5'-3')	CGTCCAGTGCCCATCAAGC
	R(5'-3')	TGAGGATGCGGAGGCGGTA
OsActin-qRT- PCR	F(5'-3')	CAACACCCCTGCTATGTACG
	R(5'-3')	CATCACCAGAGTCCAACACAA
OsLPR5-qRT- PCR	F(5'-3')	CGATGAGAATATGAGATGAAGAAGCT
	R(5'-3')	CGCACCAGTTTATGACTAGCAAA
OsPT1-qRT- PCR	F(5'-3')	CGCTTCCGTACGAGTGGTAGT
	R(5'-3')	GGTTCTTTCAAATCCAGGGAAA
OsPT2-qRT- PCR	F(5'-3')	GACGAGACCGCCCAAGAAG
	R(5'-3')	TTTTCAGTCACTCACGTCGAGAC
OsPT4-qRT- PCR	F(5'-3')	TTCTGCTAGTGTACCAAACAAAATTACA
	R(5'-3')	GTAAGTGGCATTTATAATATCAACAGTAACC
OsPT6-qRT- PCR	F(5'-3')	CCGCCCCTGCAAACTGTA
	R(5'-3')	GAACTGGCGGTTTCTTCGAT
OsPT8-qRT- PCR	F(5'-3')	AGAAGGCAAAAGAAATGTGTGTTAAAT
	R(5'-3')	AAAATGTATTCGTGCCAAATTGCT
OsPHO1;2-qRT- PCR	F(5'-3')	CGCCTAGCATGGACTGAGAGTGT
	R(5'-3')	GTTCACGGAATGGTAATGGGACA
Cis-NAT$_{PHO1;2}$-qRT- PCR	F(5'-3')	GGCTACTAGCTTGGTGCTCTTCTCC
	R(5'-3')	CTCGGAGATGAGCTTCTGCT

Supplemental Table S2. The Primers used for constructs for CRISPR/Cas9 mutation of *OsLPR5* and identification of mutants

Gene	Sequences
OsLPR5-SP1_F	ggcaGCAGCTGGCCGCTGTGTTGT
OsLPR5-SP1_ R	aaacACAACACAGCGGCCAGCTGC
OsLPR5-SP2_F	ggcaGCTGCAGACAGCGACGTTCC
OsLPR5-SP2_ R	aaacGGAACGTCGCTGTCTGCAGC
Cas9-PCR_F	ACAAGGGCAGGGATTTCG
Cas9-PCR_R	ACTGGTGGATGAGGGTGGC
OsLPR5-PCR1_F	CCTTTAATTCATTATCCTTTGC
OsLPR5-PCR1_R	ATTTTCCCGTAATCACAA
OsLPR5-PCR2_F	CATTTTCAACCCAAGACA
OsLPR5-PCR2_R	TGGGGTAGGTGTAGGTCTT
